# Evidence gaps on weight gain in people living with HIV: a scoping review to define a research agenda

**DOI:** 10.1186/s12879-023-08174-3

**Published:** 2023-04-14

**Authors:** Giovanni Guaraldi, Paolo Bonfanti, Antonio Di Biagio, Andrea Gori, Jovana Milić, Paola Saltini, Francesco V. Segala, Nicola Squillace, Lucia Taramasso, Antonella Cingolani

**Affiliations:** 1 HIV Metabolic Clinic, University of Modena and Reggio Emilia, Modena, Italy; 2 Infectious Diseases Unit, Fondazione IRCCS San Gerardo dei Tintori, Monza, Italy; 3 University of Milano-Bicocca, Milan, Italy; 4 Infectious Diseases Unit, Ospedale Policlinico San Martino, University of Genova, Genova, Italy; 5 Infectious Diseases Unit, Fondazione Istituto di Ricovero e Cura a Carattere Scientifico (IRCCS) Ca’ Granda Ospedale Maggiore Policlinico, Milan, Italy; 6 Department of Pathophysiology and Transplantation, University of Milan, Milan, Italy; 7 Infectious Diseases Unit, Fondazione Policlinico Universitario A. Gemelli-Università Cattolica Del Sacro Cuore, Rome, Italy

**Keywords:** Antiretroviral therapy, HIV, Research agenda, Weight gain

## Abstract

**Background:**

Combined antiretroviral therapy (cART) dramatically improved survival in people living with HIV (PLWH) but is associated with weight gain (WG), raising concern for a possible obesity epidemic in PLWH. This scoping review aims to identify the gaps in the existing evidence on WG in PLWH and generate a future research agenda.

**Methods:**

This review was conducted according to the methodology for scoping studies and reported according to the PRISMA Extension for Scoping Review checklist. Articles published in English in the last 10 years indexed in Pubmed, WHO Global Index Medicus, or Embase were searched using specific queries focused on WG in PLWH.

**Results:**

Following the selection process, 175 included articles were reviewed to search for the available evidence on four specific topics: (I) definition of WG in PLWH, (II) pathogenesis of WG in PLWH, (III) impact of ART on WG, (IV) correlation of WG with clinical outcomes. A summary of the data enabled us to identify gaps and clearly define the following research agenda: (I) develop a data-driven definition of WG in PLWH and define noninvasive assessment methods for body weight and fat composition; (II) further investigate the interaction between HIV/cART and immunity, metabolism, and adipose tissue; (III) establish the specific role of individual drugs on WG; (IV) clarify the independent role of WG, cART, HIV, and metabolic factors on clinical events.

**Conclusions:**

The proposed research agenda may help define future research and fill the knowledge gaps that have emerged from this review.

**Supplementary Information:**

The online version contains supplementary material available at 10.1186/s12879-023-08174-3.

## Introduction

Since the beginning of the HIV epidemic, it was clear that both HIV and antiretrovirals (ARVs) contributed to body composition changes in people living with HIV (PLWH). The “slim disease” was the first name given to AIDS to identify the main clinical presentation of the wasting syndrome affecting PLWH [1]. The use of combined antiretroviral therapy (cART) has dramatically improved the survival of PLWH. However, earlier cART medications were implicated in causing lipodystrophy, characterized by peripheral subcutaneous lipoatrophy and central/abdominal lipohypertrophy, and increased the risk of type 2 diabetes mellitus (T2DM) and cardiovascular disease (CVD).

Current cART, in particular integrase strand transfer inhibitors (INSTIs), are not related to peripheral lipoatrophy but are associated with weight gain (WG), which raises concern for a possible obesity epidemic in PLWH. Recently, tenofovir alafenamide (TAF), independent of INSTI use, has also been linked to WG [2–7].

The objective of this scoping review was to identify gaps in the existing evidence on WG in PLWH and to generate a research agenda on body composition changes in PLWH. Specifically, the following research questions (RQ) were addressed: (I) what is the available evidence/consensus on the definition of WG in PLWH; (II) what is the available evidence on pathogenesis; (III) what is the available evidence on the impact of ART on WG; (IV) what is the available evidence on the correlation between WG and clinical outcomes.

## Methods

This scoping review was conducted according to the methodological framework developed by Arksey and O’Malley [8] and refined by Levac and colleagues [9]. Reporting was performed according to the PRISMA Extension for Scoping Review checklist (Supplemental Digital Content 1), with no registration on any specific database for scoping reviews [10]. Articles published in English in the 10-year-period 2011–2021 indexed in Pubmed, WHO Global Index Medicus, or Embase were searched using specific queries focused on WG in PLWH. The search was performed on 31st October 2021. A search update was performed during October 2022, with the inclusion of 9 new records. The full search strategy and the scoping review protocol are detailed in Supplemental Digital Content 2.

## Results

### Selection of studies

**Fig. 1:**
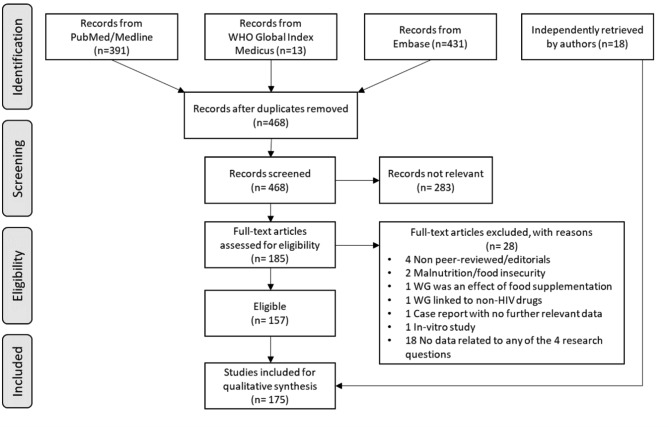
PRISMA flow diagram

Initial database extraction yielded 835 results (468 after duplicate removal). Of these, 283 were excluded, and 185 full-text articles were assessed for eligibility, which led to the inclusion of 157 articles for qualitative synthesis. During manuscript writing, additional 18 recent relevant articles were retrieved by authors (Fig. 1).

### Study characteristics

Among the 175 included articles, there were 139 original research articles, 23 reviews and 6 systematic reviews, 6 congress abstracts/presentations and 1 report from the European Medicines Agency (Supplemental Digital Content 3). Unsurprisingly, most covered aspects related to the role of cART in WG, while other areas were less well represented (Table 1).

**Table 1:** Topics of interest and distribution among included articles

			No. studies (%)
**RQ1**	Definition of WG in PLWH	WG definition used/metrics considered (ORA only)	119 (76%)
		Body composition and distribution of fat	35 (22%)
**RQ2**	Pathogenesis of WG in PLWH		108 (69%)
**RQ3**	Impact of ART on WG		142 (90%)
**RQ4**	Correlation of WG with clinical outcomes among PLWH		89 (57%)

ART: antiretroviral therapy; ORA: original research articles; PLWH: people living with HIV; RQ: research question; WG: weight gain

### RQ1. What is the available evidence/consensus on the definition of WG in PLWH?

Current literature regarding WG in PLWH suggests a definition criterion evaluating either a continuous measure of weight or an arbitrary cut-off such as a 5% weight increase or a body mass index (BMI) increase of 7%. The former cut-off of 5% is derived from recommended lifestyle interventions for weight loss as treatment of cardiometabolic conditions in the general population [11]. The latter has been used to describe the toxicities of antipsychotic drugs [12]. A recently published paper from Modena HIV Metabolic Clinic (MHMC) analyzed 54,826 weight assessments to identify WG cut off associated with the incidence of insulin resistance. A weight increase by 1% reduced the total protective effect of INSTI by 21.1% over 1 year of follow-up, which identifies a 5% weight increase as a clinically meaningful weight gain definition [13].

#### WG definition

We searched the WG definition employed in published articles on PLWH. Of the 157 included articles, we assessed 119 original research articles with relevant data on the definition of WG for this subtopic. WG relied on the assessment of weight in 52 (43.7%) studies, of BMI in 28 (23.5%) studies, and both in 37 (31.1%) studies, while other definitions such as fat gain were used in 2 (1.7%) studies only. In most papers, WG was defined as the absolute or percentage change in weight or BMI from baseline (76 studies; 63.9%), while 17 (14.3%) studies used an arbitrary pre-defined criterion, and 26 (21.8%) studies used both criteria. BMI categories of overweight and obesity were considered in 21 (17.6%) studies, whereas 17 (14.3%) studies considered a WG cut-off of 5–10%, and 11 (9.2%) studies used different definitions. The main issues related to the definition of WG in these studies are discussed below.

Most studies do not evaluate WG using a standardized follow-up period but rather measure the linear increase in weight or BMI over time. However, observational data shows that in PLWH, weight increases annually due to aging, but a more rapid increase may be observed in the first 6–12 months of therapy, particularly in people starting or switching to INSTIs. After this period, the WG trend may or may not return to parallel that observed in the general population [6]. The choice to use the absolute or percentage increase in weight or BMI is arbitrary, and the result depends mainly on the absolute value of the weight or BMI at the time of initiation or change of cART. None of the definitions mentioned above appear to be data-driven or validated in PLWH, and debate continues on the best clinical outcome measure to define clinically meaningful WG. Furthermore, WG should be considered in the context of relevant anthropometric changes that differ in ethnicity, sex, and age. For example, traditional BMI cut-offs underestimate cardiometabolic risk in the Asian population [14]. Finally, it remains unclear whether WG is synonymous with fat gain.

#### Body composition and distribution of fat

Although BMI may be a convenient and simple index to monitor the increasing prevalence of obesity at the population level, studies have shown that obesity defined by BMI is remarkably heterogeneous [15], and people with similar BMI can have substantially different comorbidities [16]. Data from several epidemiological studies over the past 30 years have shown that ectopic fat, namely visceral adipose tissue (VAT), epicardial adipose tissue, and liver fat, are independent markers of morbidity and mortality, and that the accumulation of abdominal subcutaneous adipose tissue (SAT) is a weak indicator of CV risk [17, 18]. Dual-energy X-ray absorptiometry (DEXA), computed tomography (CT), and magnetic resonance imaging (MRI) should be used to assess fat and lean mass in both observational and clinical trials in order to understand the WG phenomenon better. Among the 119 original research articles analyzed for RQ1, we identified 9 (7.6%) studies that assessed body composition with DEXA and 8 (6.7%) studies that assessed VAT components with CT or MRI. A milestone in this research area was the FRAM2 study [19]. In this study, the association between regional and total musculoskeletal tissue and adipose tissue (AT) was determined by MRI after 5 years of follow-up. After multivariate adjustments, lower arm skeletal muscle, lower leg skeletal muscle, and upper VAT were independently associated with increased mortality. The ADVANCE study included an analysis of VAT by DEXA at 96 weeks of follow-up [20]. This study compared the efficacy and safety of two first-line cART regimens (DTG + emtricitabine and either tenofovir disoproxil fumarate [TDF] or tenofovir alafenamide [TAF]) with a third regimen (efavirenz + emtricitabine + TDF). In all treatment groups, the increase in fat mass was higher than lean mass and was distributed between trunk and limbs, and a similar increase in VAT and SAT was observed. The increase in VAT between baseline and week 96 was higher in women than men, although the difference was not statistically significant, and was higher in the TAF group regarding changes in mass and volume (p < 0.001 for all comparisons) [21]. The ACTG A5260s randomized clinical trial including 234 PLWH showed that greater trunk, limb, VAT, and SAT, assessed by CT, were associated with insulin resistance and increased systemic inflammation, implying a strong relationship between fat deposits and hard metabolic outcomes over 96 weeks of follow-up [22]. Most studies evaluating the relationships between AT and health outcomes focus on the amount and distribution of AT [19, 23]. However, the quality of AT can also be indirectly assessed by measuring its density (in Hounsfield units, HU) on CT [24, 25]. As fat increases, the AT deposits can expand by hyperplasia (generation of new adipocytes of similar size), in which the density remains stable, or hypertrophy (existing adipocytes become lipid-engorged), in which the density decreases [26, 27]. Conversely, with wasting, the adipocytes become smaller and contain fewer lipids, which is reflected by an increase in density [28, 29]. Inflammation and fibrosis of the AT can also cause an increase in its density [24], which may be a marker of AT dysfunction [29, 30] and potentially increased cardiometabolic risk [26, 28]. On the other hand, lower VAT and SAT density (which reflects fat engorgement of adipocytes, likely without progression to significant fibrosis) was associated with increased cardiometabolic risk, higher levels of circulating leptin, and lower levels of adiponectin [24, 31]. Another study that included 418 PLWH who switched to INSTIs showed that a higher increase in BMI over the 4-year follow-up was associated with a lower VAT density, suggesting improved fat quality [32].

### RQ2. What is the available evidence on the pathogenesis of WG in PLWH?

The complex, interrelated mechanisms in WG associated with cART in PLWH are not yet fully understood.

#### Role of HIV and/or inflammation

HIV-associated wasting accompanying advanced CD4 cell depletion is characterized by an increase in basal metabolism, which can increase further during opportunistic infections. Anorexia, secondary to the effect of elevated inflammatory markers on the hypothalamus, also occurs at this stage of the disease [33]. ART initiation reverses this metabolic state and reduces inflammation, reflecting a reduced metabolic demand in the state of controlled infection, possibly leading to WG. Several studies showed that PLWH with lower CD4 counts and higher viral loads (VL) at ART initiation gain more weight after treatment initiation [34, 35]. The Swiss HIV Cohort study showed that a lower CD4 nadir is a significant risk factor for developing general and visceral obesity; in particular, a 2-fold increased risk of general obesity was observed in patients with CD4 nadir < 100 cell/µL [36]. In the Italian SCOLTA cohort, patients in CDC stage C treated with different ARV had a higher BMI increase than patients in stage A and B [37]. The results of these studies support the hypothesis that WG may be interpreted, at least in part, as a “return to health” phenomenon, characterized by reduced or suppressed viral replication, a reduced metabolic demand by the virus, and a better control of inflammation [38]. In a study conducted among people with acute and early HIV infection, VL and CD4 nadir were not identified as risk factors for WG after ART initiation. A possible explanation for this unexpected finding is that the duration of infection could play a role in WG. Moreover, HIV-RNA and CD4 count in acute and early HIV infection have a unique dynamic that weakens their associations with weight change [39]. HIV-associated immune activation persists despite suppressive ART. Innate and adaptive immunity cells are also located in the stromal vascular fraction of AT, where they modulate adipocyte energy storage and function, and inflammation [35]. Macrophages accumulate in AT with progressive WG. Adipocytes and macrophages produce the monocyte chemoattractant protein 1 (MCP-1), which drives the increase in TNFα, IL-6, IL-12, and IL-23. A study of gluteal fold AT showed similar macrophage density between PLWH and HIV-negative controls but higher IL-6, IL-8, IL-12p40, and monocyte inflammatory protein 1α (MIP1α) levels in the HIV-positive population [40]. In a French cohort of patients with normal weight at ART initiation, who achieved virologic suppression, most inflammatory biomarkers, namely CXCL8, sTNFR1, sTNFR2, and sCD163, were significantly higher in patients who became overweight or obese [41]. These inflammatory biomarkers tend to decrease with ART in the setting of a controlled infection. Accordingly, also in two large randomized ACTG trials A5202 and A5257, it was observed that among non-obese persons, higher pre-treatment immune activation markers significantly associated with WG in course of ART, while WG attenuated the decline in several immune activation markers following ART initiation [42]. Furthermore, it is known that lipopolysaccharide (LPS), which is increased in PLWH because of the breach of the gut barrier, facilitates inflammation and fibrosis of AT [43]. In animal models, LPS triggers gains in visceral AT and AT inflammation mediated via LPS’s coreceptor, CD14 [44]. LPS also stimulates the proliferation of pre-adipocytes through a CD14‐dependent mechanism, possibly through activation of JAK/STAT and AMPK via cytosolic phospholipase A2 [45].

Moreover, obesity and HIV are both associated with a loss of gut microbial diversity and a deleterious metabolome [46]. The composition and biodiversity of the gut microbiota have been found to correlate with changes in weight in experimental animals and, to a lesser degree of evidence, also in humans [47, 48]. The microbiota, in turns, may influence levels of immunoactivation and translocation of microbial products, as well as being an active participant in the process of metabolization and digestion of certain nutrients [47].

WG is associated with increased transforming growth factor-β that triggers a pro-fibrotic process to limit adipocyte hypertrophy. However, ectopic fat deposition occurs in sites such as the liver and skeletal muscle, worsening the proinflammatory state, metabolic dysregulation, and tissue hypoxia. Different studies documented a reduction of AT fibrosis and several inflammatory biomarkers during cART [43].

#### Role of host-related factors

Current literature regarding WG in PLWH also focuses on different host-related risk factors, such as age, sex, country of origin, and BMI at ART initiation. When considered, older age is often associated with a higher risk of WG [38, 49, 50]. However, a recent study showed that significant WG was not observed in geriatric PLWH who switched to a dolutegravir (DTG)-based regimen [51]. It cannot be excluded that the stability of weight over time in this study was rather the result of a relative increase in fat mass in a population with a high rate of lean mass loss, associated with age-related sarcopenia [52]. On the other hand, few studies have been conducted in children and adolescents, where WG during the growth stages is a desired factor and usually precedes height increase, while growth deficits often persist in young PLWH even after ART initiation, especially in low income countries [53]. However, in children and adolescents, no significant differences in WG trajectories have been reported after switching from other regimens to INSTIs [54–56] although not all studies agree [57].

The country of origin of PLWH may also impact WG. For example, BMI was lower in patients living in low and low-middle income countries in Africa and Asia and higher in patients from middle-high- and high-income countries in a systematic review and meta-analysis of BMI changes among treatment-naïve patients who started ART [58]. Similarly, in the general people living without HIV, rates of overweight and obesity are considerably different depending on country and culture of origin, with north American men and South African women being the population with the highest prevalence of obesity, further increasing in recent years [59]. Many studies focusing on WG in course of ART pointed out how black people could be more prone to WG in certain settings, however behavioral and cultural differences are poorly investigated in most studies and might perhaps explain this data, also in light of the fact that WG is desired rather than unwelcomed by some patients for cultural reasons [60, 61]. Moreover, people from the same ethnicity may have different prevalence of overweight at baseline and during ART treatment depending on the country where they live, for instance in Latin Americans living in Latin America, the prevalence of obesity has been reported 5% before ART and 13% after three years of ART, while the corresponding frequencies for Latin Americans living in US or Canada were 15 and 22% [62], suggesting that lifestyle, more than ethnicity, may be relevant for WG. Therefore, socioeconomic status including marital status could strongly impact on WG in PLWH [41, 63], but it is an under reported neglected measure in HIV scientific literature. This may be a consequence of unmeasured various different cultural factors, or unobserved data on diet, physical activity and job, which have a significant impact on WG.

Sex also needs to be considered in evaluating host factors determining WG in PLWH on ART. Several studies highlighted an increased risk of WG in women compared with men [4, 34, 64, 65], but this is not confirmed worldwide, and there are conflicting results [38, 66]. Moreover, this is still largely unexplored in transgender PLWH.

The reason for the different incidence of WG in male and female are still under investigation. However, this difference reflects the epidemiology observed in the general population, where obesity is more prevalent in women than men in both developed and developing countries, and increase with age [67]. As estrogen promotes brown adipocyte differentiation while suppressing white adipose differentiation, some authors have hypothesized that, in the context of treated HIV infections, some ARVs might be correlated to higher effect on WG in women by interacting with this pathway. According to previous study, INSTIs may interrupt adipose function via inhibition of estrogen action, as it was found that estrogen-mediated pathway was blocked by DTG by 70% and DTG administration to female mice inhibited oxygen consumption and energy expenditure by 15% without affecting food consumption [68]. Moreover, different inflammatory patterns and trajectories of inflammatory markers in course of ART have been observed in female compared to male PLWH, suggesting once again a possible interplay between inflammation and WG also in such context [42].

Finally, the role of body weight before ART initiation in determining WG is controversial in the literature. A lower baseline weight is generally considered to be a risk factor for WG [5, 37, 38, 49, 66, 69–72]. However, a cohort study of PLWH found no significant difference in WG when stratified by baseline weight [66]. In a French cohort of normal-weight patients at ART initiation, patients who retained a normal weight had a lower baseline BMI compared with those who became overweight or obese [41]. In addition, in switch studies, it appears that PLWH with higher BMI gain more weight, underscoring how, after the initial impact of ART on the virus and on an initial “return to health”, it may be lifestyles that impact more on the WG outcome [73, 74].

Another variable frequently associated with a higher risk of WG is smoking. In the Swiss HIV cohort study, smoking was significantly associated with general and abdominal obesity [36]. As smoking increases inflammation, it could also increase WG per se. However, smoking is not analyzed as a risk factor in most studies [63].

#### Possible pathogenetic mechanisms related to antiretroviral drugs

Many studies conducted in vitro in recent years have tried to find a biologically plausible explanation for the increased WG observed in course of some ARVs. One of the first hypothesis had been that INSTIs and, in particular, dolutegravir, could exert an inhibitory effect on α-melanocyte-stimulating hormone (α-MSH), an anorexigenic neuropeptide, by binding of melanocortin 4 receptor, which may interfere with the regulation of food intake and lead to obesity [75]. However, this hypothesis was subsequently tested in vitro, founding that, for all INSTIs, drug concentrations substantially greater than clinical exposure are required for antagonism of the melanocortin-4 receptor to occur [76].

Another possible mechanism advocated to explain the weight gain is the interference of ARVs in the process of adipogenesis and energy expenditure of adipose tissue. In vitro data showed that drugs little associated to weight gain such as efavirenz and, to a lesser extent, elvitegravir, could alter adipocyte differentiation and induce pro-inflammatory cytokines, in a concentration-dependent manner, delaying acquisition of adipocyte morphology and reducing the expression of adipogenesis marker genes such as PPARγ, glucose transporter GLUT4, lipoprotein lipase, and the adipokines adiponectin and leptin [77]. In contrast, raltegravir, and to a greater extent, dolutegravir, at peak concentration have been associated with elevated adipogenesis and lipid accumulation in adipocyte-differentiated adipose stem cells, suggesting these INSTIs may have a role in adipogenesis, lipogenesis, oxidative stress and insulin resistance [78]. In accordance with this hypothesis, raltegravir, dolutegravir, but also bictegravir and elvitegravir have been found to be able to increase adipogenesis in vitro, as demonstrated by the induction of higher levels of PPARɣ and C/EBP⍺ in adipocytes exposed to INSTIs compared to controls [79]. A recent study also found that PLWH on INSTIs, had reduced populations of metabolically activated CD9 + adipose tissue macrophages, that are considered metabolically beneficial, compared to that of uninfected controls (P < 0.001). Moreover, BMCs of PLWH had lower fatty acid metabolism compared to those of uninfected controls (P < 0.01) and, according to the analysis performed in murine macrophages, dolutegravir reduced lipid metabolism and increased expression of the fatty acid beta-oxidation enzyme Enoyl-CoA Hydratase, Short Chain 1 [80].

Dolutegravir has also been found to decrease oxygen consumption of preadipocytes, besides reducing, in course of adipocyte differentiation, triglyceride accumulation and adiponectin secretion, suggesting a possible additive mechanism of mitochondrial impairment [68, 81]. However, it is important to point out that all these data are derived only from in vitro studies, and there are currently no in vivo studies that correlate a molecular/cellular mechanism to the WG phenomenon, in addition to not always being confirmed the same results in different laboratory studies. Research in this field still requires an in-depth clinical field effort with longitudinal studies proving the presence or absence of the supposed mechanisms in people who gain or do not gain weight.

### RQ3. What is the available evidence on the clinical impact of ART on WG?

WG in PLWH appears to be less evident in people treated with older NRTI and NNRTIs [70, 82–85] to the extent that a protective effect has been hypothesized, although not demonstrated, for some drugs of these classes, such as EFV and TDF [86–88]. However, high exposure to these drugs may be related to less WG because of gastrointestinal symptoms inducing anorexia, lipoatrophy, or other unclear mechanisms. Indeed, in people who are extensive EFV metabolizers because of CYP2B6 polymorphisms, WG is similar to that observed with DTG [87]. Additionally, it is important to point out that, although EFV and TDF are less related to the WG phenomenon compared with the newer ARVs, on the contrary, in older studies, TDF was associated with greater WG when compared with older ARVs, such as zidovudine or other nucleoside/nucleotide reverse transcriptase inhibitors, while EFV compared with nevirapine and even with protease inhibitors [70, 89]. This could suggest how, as the tolerability of ART improves, we see greater WG, not necessarily given by a side effect, but possibly related to an absence of toxicity. Regardless of the underlying mechanism, when a patient switches from an older drug to a newer ARV, WG is often significant [90]. For instance, PIs have been associated with higher WG compared with NNRTIs [69, 91–93], but the drugs with the highest impact on WG seem to be the newer ARVs, namely INSTIs and TAF [70, 72, 73, 94–96], which have an even more pronounced effect when they are used in combination [20, 97, 98]. However, it remains challenging to establish the role of a single agent in determining WG when, by definition, ART involves the use of at least two drugs and WG is known to have a multifactorial genesis. Also, it remains to be ascertained the role of a possible switch strategy to NNRTI or older treatments to contrast the phenomenon of WG. A proposal of switching from DTG to EFV and from TAF to TDF the study participants of the ADVANCE trial who had experienced the highest amount of WG, has been recently made by the authors of the study [99]. However, the patients themselves refused this proposal, besides being a step backward toward less innovative and less tolerated therapies [99, 100]. While the removal of TAF by switching from a TAF containing triple therapy to DTG and lamivudine two-drug therapy did not impact on weight gain [101], a first study comparing weight trajectories in people continuing TAF versus switching to TDF has been recently published in a North-European retrospective cohort of 292 patients [102]. In this study, people who remained on TAF increased their weight of mean + 0.9 kg after one year, while those switched to TDF did not experience a significant weight increase during the same follow up. However the interpretation of these results is limited by the small number of participants (weight analysis at one year made on 90 PLWH on TAF and 65 PLWH on TDF) and by the retrospective design, while clinical trials evaluating the effect on weight of a switch strategy from INSTI to TAF/emtricitabine/darunavir/cobicistat (DEFINE trial, NCT04442737) or to Doravirine/Lamivudine/TDF (DeLiTE trial, NCT04665375), and from regimens containing both INSTIs and TAF to TDF/XTC/doravirine (NCT04636437), are in course and actively enrolling, to respond to the important question of which strategy could be better to face the problem.

In addition, given the lack of patients’ perspective currently reported in the published literature concerning the switch of ARVs due to WG, a proper attention to this topic should be paid in future studies. Indeed, a better evaluation of patients point of views may be of help to deepen the reason for switching or non-switching, to support the clinical decision.

#### Does TAF really contribute to body WG?

Randomized controlled trials, including INSTI-based regimens in naïve PLWH, demonstrated a greater WG in patients starting TAF versus TDF that varied significantly (from a maximum of 6 kg in 48 weeks to a minimum of 3.6 kg in 96 weeks) largely depending on sex and race differences in the trial populations [20, 50, 70, 103, 104]. In addition, the AMBER study identified a greater WG in naïve PLWH taking TAF than those taking TDF (2 kg versus 1 kg in 96 weeks), both in combination with darunavir/cobicistat [105]. Data from observational studies also confirmed a possible contributing role of TAF in WG. In a recent analysis of the RESPOND cohort, including 14,703 ART naïve and ART-experienced PLWH, use of TAF (compared with lamivudine) was independently associated with a > 7% increase in BMI [72]. These observations also apply to studies that only consider PLWH with HIV RNA < 50 copies/mL, where a greater WG is generally observed after switching to TAF compared to TDF, but not to abacavir [7, 73, 104]. One of the hypotheses is that the WG reported in switch studies may be due, at least in part, to the loss of the protective effects of TDF and/or EFV rather than the effects of TAF [104]. This was also observed in the switch study to BIC/TAF/FTC [106]. In this study, weight change differed by previous NRTIs (+ 2.2 kg [F/TDF] and + 0.6 kg [F/TAF], p < 0.001), while no differences were found between BIC/TAF/FTC and DTG + TAF/FTC. Finally, in a pre-exposure prophylaxis (PrEP) study in people living without HIV taking TAF or TDF along with FTC, there was a difference of + 1 kg in the TAF/FTC arm [107]. Therefore, whether TAF promotes WG or TDF reduces weight remains to be ascertained.

#### What is the role of INSTIs?

In recent studies, INSTIs have been associated with an excess WG compared with other ARV classes [90]. However, differences exist among individual INSTIs and different study populations, with higher WG observed with DTG or BIC in naïve PLWH initiating first-line ART [20, 70, 91] than in switch studies, where results are somewhat conflicting [37, 56, 106, 108–111]. Moreover, WG was higher in studies performed in African and North American populations [20, 91, 112], and smaller in European cohorts [37, 51, 56, 108, 110].

The data on individual INSTIs are also controversial. Initially, DTG and BIC seemed to have the highest impact on WG, but further studies have also suggested a relevant role of RAL and EVG. DTG was the first drug to be associated with WG [4, 91, 113]. In a retrospective observational cohort study [114] in 1152 ART-naïve PLWH, adjusted mean WG after 18 months was significantly higher with DTG (6.0 kg, 95% CI, 4.2–7.8) compared with NNRTIs (2.6 kg, 95% CI, 1.5–3.6) or EVG (0.5 kg, 95% CI, -1.0–2.0), while no significant difference was found compared with RAL (3.4 kg, 95% CI, 1.8–5.0) or PIs (4.1 kg, 95% CI, 3.2–5.0) [114]. Similar results were then confirmed in the larger observational NA-ACCORD study [91], where PLWH starting INSTI-based regimens had a mean estimated 5-year WG of + 5.9 kg, compared with + 3.7 kg with NNRTIs and + 5.5 kg with PIs. Among PWLH starting INSTIs, the mean estimated 2-year WG was + 7.2 kg for DTG, + 5.8 kg for RAL, and + 4.1 kg for EVG [91].

PLWH starting DTG in clinical trials experienced WG ranging between + 2.1 and + 7.1 kg after 96–144 weeks if ART-naive [20, 70, 112, 115, 116], and between + 0.8 and + 0.98 kg after 48–96 weeks, if ART-experienced [117, 118]. This corresponds to a modest change in BMI over time, particularly in DTG switching strategies, with an unknown impact on long-term mortality or metabolic outcomes, especially in normal-weight people.

In studies in naïve PLWH, such as the STARTMRK, RAL was associated with similar increases in BMI compared with EFV after 156 weeks [119]. In addition, BMI increased by 3.8–4.7% in ART-naïve PLWH, similarly in the three study arms (DRV/r or ATV/r or RAL), in the AIDS Clinical Trials Group (ACTG) A5260s metabolic substudy of the A5257 randomized trial [22]. However, an analysis of the whole cohort of the A5257 study, showed that the initiation of RAL was associated with higher weight/BMI increases than ATV/r or DRV/r in treatment-naïve patients with normal BMI or who were underweight but became overweight or obese during follow-up [120]. Moreover, in the large, prospective, multicohort RESPOND collaboration, which included data on both ART-naïve and experienced PLWH, both RAL and DTG were found to be associated with an increased risk of both > 7% and > 30% BMI, while no increased risk was found for EVG [72]. In a pooled analysis of ACTG A5142, A5202, and A5257 (only the latter study included a group of patients on INSTI), a significantly greater WG was observed in women than in men [64]. In the WIHS cohort, PLWH on INSTI and/or TAF with a normal BMI at baseline had a significant risk of WG [121].

In a non-inferiority study of DTG/ABC/3TC versus BIC/FTC/TAF in ART-naïve PLWH, the median WG after 96 weeks was 2.4 and 3.6 kg, respectively [122]. In another non-inferiority study of DTG + FTC/TAF versus BIC/FTC/TAF in ART-naïve persons, the median changes in body weight after 96 weeks were + 3.9 kg and + 3.5 kg, respectively [123].

Cabotegravir (CAB) is the first long-acting injectable INSTI, recently approved for use in therapy and PrEP for HIV infection. A recent study investigating WG among 177 people living without HIV who received at least one injection of CAB or placebo (134 CAB; 43 placebo) found no difference between the two study arms [124]. On the other hand, when CAB was used in switching strategies in PLWH, a similar WG was observed between CAB and comparator ARTs [125].

### RQ4. What is the available evidence on the correlation of WG with clinical outcomes?

#### Cardiovascular complications

PLWH have an increased risk of CVD compared with people without HIV, regardless of the ART regimen [126]. However, the association between CV risk and ART-associated WG is controversial. For example, observational data showed that, although increases in BMI among ART-exposed PLWH were linked to an increased risk of T2DM, such changes did not show an association with increased CV risk [127]. In this study, CVD was defined as the first event from a composite of myocardial infarction (MI), sudden cardiac death, invasive CV procedure (coronary artery bypass graft or carotid endarterectomy), or stroke [127]. In addition, an increased long-term risk for major CV events was identified in PLWH who experienced a short-term gain in BMI (within 1 year from ART initiation), especially those with a baseline BMI within 18.5 and 25 kg/m^2^ [5]. Of note, this is the only study to investigate the relationship between WG in ART-exposed individuals and CV risk.

#### Diabetes

The risk of developing T2DM is associated with BMI in the general population [128]. The Data Collection on Adverse Events of Anti-HIV Drugs (D:A:D) cohort study on 43,278 person-years (N = 9193) reported 125 diabetes events with an incidence rate ratio (IRR)/unit gain in BMI of 1.11 (95% confidence interval 1.03 to 1.21) regardless of the pre-ART BMI [5]. This suggests that the short-term gain in BMI following ART initiation appears to increase the risk of T2DM independently from baseline BMI [5]. A meta-analysis of 41 observational studies evaluating PLWH who were either ART-naive or on ART found increased fasting blood glucose levels and an increased risk of developing T2DM in those treated with ART [129]. In the REPRIEVE study including 1848 PLWH on an INSTI-based regimen, INSTI use was not associated with a difference in mean fasting glucose, even when PLWH were stratified by natal sex [130]. Another meta-analysis across 44 studies also found ART to be a risk factor for T2DM development in some, but not all, studies [131]. A study comparing Taiwanese PLWH on ART (most commonly ≥ 2 NRTIs with NNRTIs or boosted or unboosted PIs) with those not on ART showed that the risk of developing T2DM was associated with ART use [132]. The interplay between WG and ART regimens in the development of T2DM is a complex topic. Evidence is emerging on the association among WG, INSTIs, and T2DM. In PLWH switching to INSTIs, an increase in HbA1c was observed [86, 133]. In another cohort study, participants initiating INSTIs (particularly raltegravir) were more likely to develop T2DM than patients starting NNRTIs [93]. In the EMERALD trial, TAF was significantly associated with treatment-emergent T2DM compared with TDF [134]. In a retrospective analysis of the ADVANCE study data, among PLWH on INSTIs, obesity drove an increased risk of T2DM, especially in those taking TAF/FTC + DTG, with some evidence of greater CVD risk [97].

#### Metabolic syndrome

The prevalence of metabolic syndrome (MetS), characterized by central obesity and insulin resistance, is rapidly increasing in PLWH treated with cART [135]. Studies demonstrated that the incidence of MetS increases among PLWH, and its prevalence remains high after initiating ART [136, 137]. In the ADVANCE study, treatment-emergent MetS was observed, especially in the TAF/FTC + DTG arm and among women [20, 114]. However, ART is probably not the sole reason for these metabolic disorders since HIV is also likely to affect metabolism. The persistence of HIV in tissue reservoirs could synergize with some ART-enhancing metabolic disorders [138]. Mitochondrial dysfunction seems to be the most common underlying mechanism used by HIV and most ARVs to cause inflammation, insulin resistance, dyslipidemia, and lipodystrophy [139]. Findings indicate that PIs are more commonly implicated in MetS-related effects than other classes of ARVs, due to their ability to initiate many toxicities leading to MetS [138, 139]. Female gender, high BMI, and older age are major risk factors for the occurrence of MetS, and hypertriglyceridemia and low levels of high-density lipoproteins are the most common types of dyslipidemia [138].

#### Chronic kidney disease

PLWH have higher rates of chronic kidney disease (CKD), and studies have demonstrated an increased risk of CKD correlated with the use of ART [140–142]. Concerning the impact of WG on kidney function, large cohorts from Spain and the US found that PLWH with both end-stage renal disease (ESRD) and mild reduction of glomerular filtration rate (60–89 mL/min/1.73 m^2^) were more likely to have higher BMI at univariate analysis. However, when compared with PLWH with normal renal function, PLWH with reduced glomerular filtration rates had higher BMI, among other factors such as older age, female gender, and NRTI or PI ritonavir-regimens [140, 143]. Despite the relevance of the research question, to our knowledge, so far no study directly assessed the issue on how increased WG impacts renal function among PLWH.

#### Liver disease

Non-AIDS-defining comorbidities in an aging population such as PLWH include non-alcoholic fatty liver disease (NAFLD), whose prevalence in the general population is estimated to be around 25%, and NAFLD is related to increasing weight as well as the development of MetS. In PLWH, the prevalence of NAFLD ranges between 13% and 73% [144, 145]. Specific HIV-related risk factors (e.g., persistent immune activation and ARV) may alter the course of liver disease [146]. Consistently with the return-to-health phenomenon, WG shortly after ART initiation was associated with improved survival in PLWH, particularly those with more advanced disease [90]. INSTIs and TAF are also associated with an increased risk of developing hepatic steatosis [147].

## Discussion

This scoping review attempted to address four questions regarding WG in PLWH. For each, our literature interpretation only partially answered the question but helped us to define the following research agenda with improved clarity.

### Definition of WG in PLWH

We believe that the complexity of the correlations between fat quantity and distribution and fat and lean mass and their implications at the clinical level cannot be resolved by simply evaluating the change in weight or BMI. We, therefore, suggest that the following non-exhaustive research program on how to appropriately define and measure WG in PLWH is addressed in future research: (I) develop a clinically meaningful data-driven definition of WG; (II) define methods of non-invasive assessment of weight and body composition to be used in clinical practice; (III) recognize the density of fat and lean tissue as a measure of their quality and function; (IV) study the interrelationship between fat and lean tissue and bone in metabolic homeostasis.

### Pathogenesis of WG in PLWH

Further research is needed to understand how HIV infection and ART influence the complex interaction between AT, innate and cellular immune function, and metabolism. For host-related factors, meta-analyses may help resolve the observed conflicting results. Moreover, in future research, all relevant host-related factors must be recorded and considered while adjusting analyses for potential confounders (including smoking and socioeconomic status, which are often unreported), to better identify, and to carefully manage with new intervention strategy PLWH at higher risk of WG.

### The impact of ART on WG

Further research is needed to establish how TAF and INSTI contribute to WG in PLWH, possibly highlighting the specific role of TAF by stopping TDF and/or EFV. Moreover, the impact of non-pharmaceutical intervention protocols against WG, like diet and physical exercise, should be considered.

### WG impact on clinical outcomes

Further studies are required to investigate: (I) the independent role of WG on clinical events compared with other metabolic factors and ART; (II) the role of specific HIV-related factors versus generic factors in the development of WG-related clinical events; (III) the association between ART and WG as a driver of clinical events.

### Limitations

This work presents the intrinsic limitations of the scoping reviews due to the related methodology. The evaluation of risk of bias of included studies is not performed. In addition, despite some indications on WG in children and adolescents are reported in the text, the authors are aware that the inclusion criteria referred only to adults WG. The comment on WG in children and adolescent has to be interpreted as a pure consideration of the state of the art.

Despite not directly adding scientific knowledge to the field, this work highlights the current knowledge gaps in the topic thus providing a future research agenda to fill the unmet requirements.

## Conclusion

This review illustrates knowledge gaps regarding WG in PLWH for each RQ, despite the rapidly growing number of articles on this topic. The literature is mainly focused on the role of drugs. However, the absence of a shared clinically meaningful definition of WG impairs comparability among studies and the opportunity to derive strong clinical guidance. Therefore, we believe our research agenda may help define future research and fill the knowledge gaps that have emerged from this review.

### Electronic supplementary material

Below is the link to the electronic supplementary material.

Supplementary Material 1

Supplementary Material 2

Supplementary Material 3

## Data Availability

All data generated or analysed during this study are included in this published article and its supplementary information files.
