# Supplementary material for: Evidence gaps on weight gain in people living with HIV: a scoping review to define a research agenda
Source: BMC Infect Dis. 2023 Apr 14;23:230. doi: 10.1186/s12879-023-08174-3 (PMC10103467; doi:10.1186/s12879-023-08174-3)
Supplement: Supplementary file 3 — Supplementary Material 3 [file 12879_2023_8174_MOESM3_ESM.pdf]

Weight gain in people with HIV who are undergoing antiretroviral therapy

Scoping review protocol

INTRODUCTION

Since the introduction of combined anti-retroviral therapy (cART) in the mid-1990s, effective virological suppression has dramatically improved prognosis and survival of people living with HIV (PLWH). However, concerns of cART-associated metabolic complications have emerged. Earlier cART medications were implicated in causing lipodystrophy, characterized by peripheral subcutaneous lipoatrophy and central/abdominal lipohypertrophy and shown to increase risk of type 2 diabetes mellitus (T2DM) and atherothrombotic cardiovascular disease (CVD). More recently such medications are avoided, but longitudinal studies have shown that cART is associated with weight gain, particularly with the development of visceral adiposity, with increased rates of incident CVD and T2DM [**Lake 2017, Kumar 2018**]. PLWH are already at higher risk of CVD *per se*, when compared with the HIV- uninfected population, and therefore, the control and correction of modifiable risk factors for CVD and metabolic disease, including body mass index (BMI) and weight, are of major importance [**Taramasso 2017].**

Aim of the study

The primary objective of this scoping review is to summarize the evidence gaps on weight gain in people with HIV who are undergoing antiretroviral therapy.

Research questions

The study will address the following questions:

1. What is the available evidence/consensus on the definition of weight gain in PLWH?
2. What is the available evidence on the pathogenesis of weight gain in PLWH?
3. What is the available evidence on the correlation of weight gain with clinical outcomes among PLWH?
4. What is the available evidence on clinical management of weight gain in PLWH?

METHODOLOGY

Study design

To clarify key concepts/definitions in the literature and identify and analyse knowledge gaps are among the traditional objectives of scoping reviews [**Munn 2018**], thus this methodology was chosen.

A scoping review will be conducted according to the methodological framework developed by Arksey & O'Malley and refined by Levac et al. [**Arksey 2005, Levac 2010**].

Reporting will be performed according to the PRISMA Extension for Scoping Review checklist [**Tricco 2018**].

Search strategy

Published articles indexed on Pubmed, WHO Global Index Medicus and Embase are searched using the following queries.

Pubmed

("weight gain" OR "weight increas*" OR "weight change*" OR "BMI increas*" OR "body mass index increas*" OR "BMI change*" OR "body mass index change*") AND (hiv[tiab] OR "HIV"[Mesh] OR "Acquired Immunodeficiency Syndrome"[Mesh] OR "HIV infections"[Mesh] OR "Acquired Immunodeficiency Syndrome"[tiab] OR "Human immunodeficiency virus"[tiab])

Filters applied: Full text, Journal Article, in the last 10 years, English.

WHO Global Index Medicus (https://www.globalindexmedicus.net/)

("weight gain" OR "weight increase" OR "weight increased" OR "weight increasing" OR "weight increases" OR "weight change" OR "weight changes" OR "weight changed" OR "BMI increase" OR "BMI increased" OR "BMI increases" OR "body mass index increase" OR "BMI change" OR "body mass index change" OR "BMI changed" OR "body mass index changed" OR "BMI changes" OR "body mass index changes") AND (hiv OR aids OR "Acquired Immunodeficiency Syndrome")

Filters applied: In the last 10 years, English.

Embase

#1

('weight gain':ti,ab,kw OR 'weight increas*':ti,ab,kw OR 'weight change*':ti,ab,kw OR 'bmi increas*':ti,ab,kw OR 'body mass index increas*':ti,ab,kw OR 'bmi change*':ti,ab,kw OR 'body mass index change*':ti,ab,kw) AND ('human immunodeficiency virus'/exp OR 'acquired immune deficiency syndrome'/exp OR 'hiv':ti,ab,kw OR 'Human immunodeficiency virus':ti,ab,kw OR 'Acquired Immunodeficiency Syndrome':ti,ab,kw)

#2: #1 NOT ([conference abstract]/lim OR [conference paper]/lim OR [conference review]/lim)

#3: #2 AND [english]/lim AND [2011-2021]/py

Eligibility criteria

2 independent reviewers will screen title/abstracts for relevance.

Inclusion criteria:

- Specific reference to weight gain in adults (aged 18 years old or above) living with HIV/AIDS
- Published in the last 10 years
- Full text available in English

Exclusion criteria:

- In-vitro / non-human studies
- Non-peer-reviewed articles (e.g. Conference abstracts / posters / conference paper / conference review)
- Weight change identified as a side effect of non-HIV drugs (e.g. TB drugs)
- Focus on malnutrition/food security
- For original research articles: no results stratified/specific for PLWH available (e.g. population with a percentage of PLWH included but only data for the whole population available, without stratification according to HIV infection)

Full-texts will be then assessed to check relevance to the 4 research questions.

Regarding research question #4, and specifically the role of drugs, the following treatments will be ignored since they are not used anymore or they are used only in very specific settings:

tipranavir (TPV), **maraviroc** (MVC), **indinavir** (IDV), **enfuvirtide** (ENF), **etravirina**, (ETR), **saquinavir** (SQV), **ritonavir**, **zidovudina** (AZT), **efavirenz** (EFV), **fosamprenavir** (FPV), **nelfinavir** (NFV), **didanosina** (Ddi), **nevirapina** (NVP), **stavudina** (d4t), **Combivir®** (AZT + 3TC), **lopinavir/r** (LPV/r)

Atripla® (TDF+FTC+EFV), **Trizivir®** (AZT+3TC+ABC)

Data charting

A data extraction Excel sheet will summarize relevant data from the included studies, organizing the information according to the 4 areas covered by the research questions.

The following fields are recorded:

- Title
- Objective
- Authors
- Journal
- Year
- Full-text - Relevant?
- If no, reason
- Primary literature/ Systematic review/ Review
- If primary:
    - Type of study

    - N of PLWH included
    - Does the group have any eligibility criteria?
    - If yes, specify as described in the paper
    - Ethnicities (use % if possible)
    - "Sex
    - (Male %)"
    - Age (median or average, if no median available)
    - Age (interquartile range, IQR, or Std deviation, SD)
    - ART naïve (%)
    - Lenght of study (mo)
    - Baseline weight (kg)
    - Weight IQR or SD
    - Baseline BMI (kg/m2)
    - BMI IQR or SD
    - Comorbidities (% if possible)
- 1- Definition of WG used
- 1- Body composition and distribution of fat
- 2- Data on the pathogenesis of WG in PLWH (role of HIV, host factors, inflammation and infl. biomarkers)
- 3- Data on the correlation of WG with clinical outcomes among PLWH (including PRO)
- 4- Clinical management of WG in PLWH - role of drugs
- 4- Clinical management of WG in PLWH - intervention protocols against WG
- 4- Clinical management of WG in PLWH - monitoring of WG

Data analysis

The main findings from the included studies will be presented narratively, highlighting existing knowledge gaps.

Being a scoping review, risk of bias assessment will not be performed.

As initially mentioned, the focus of this scoping review will be on weight gain, thus evidence of weight loss will not be discussed, unless when mixed/uncertain/contrasting results for drugs are found among different studies, otherwise the omission of the studies where weight loss is observed would result in a selection bias.

Limitations

Consistently with other scoping reviews, risk of bias or other assessment of included studies is not performed. Moreover, it does not directly advance scientific knowledge but it provides a research agenda to fill the knowledge gaps that have emerged from this review.

References

Arksey H, O'Malley. Scoping studies: towards a methodological framework. Int J Soc Res Methodol 2005 Feb;8(1):19–32.

Kumar S, Samaras K. The Impact of Weight Gain During HIV Treatment on Risk of Pre-diabetes, Diabetes Mellitus, Cardiovascular Disease, and Mortality. Front Endocrinol (Lausanne). 2018 Nov 27;9:705. doi: 10.3389/fendo.2018.00705.

Lake JE. The Fat of the Matter: Obesity and Visceral Adiposity in Treated HIV Infection. Curr HIV/AIDS Rep. 2017 Dec;14(6):211-219. doi: 10.1007/s11904-017-0368-6.

Levac D, Colquhoun H, O'Brien KK. Scoping studies: advancing the methodology. Implement Sci 2010;5(1):69.

Munn Z, Peters MDJ, Stern C et al. Systematic review or scoping review? Guidance for authors when choosing between a systematic or scoping review approach. BMC Med Res Methodol 18, 143 (2018). https://doi.org/10.1186/s12874-018-0611-x

Taramasso L, Ricci E, Menzaghi B, et al. Weight Gain: A Possible Side Effect of All Antiretrovirals. Open Forum Infect Dis. 2017 Nov 3;4(4):ofx239. doi: 10.1093/ofid/ofx239.

Tricco AC, Lillie E, Zarin W, et al. PRISMA Extension for Scoping Reviews (PRISMAScR): Checklist and Explanation. Ann Intern Med 2018;169(7):467–74.
